# Cannabis and Psychosis: Are We any Closer to Understanding the Relationship?

**DOI:** 10.1007/s11920-019-1044-x

**Published:** 2019-06-04

**Authors:** Ian Hamilton, Mark Monaghan

**Affiliations:** 1 Health Sciences, University of York, York, UK; 2 Social Policy, University of Birmingham, Birmingham, UK

**Keywords:** Cannabis, Psychosis, Schizophrenia, Diagnosis, Cannabis use disorder, Sex

## Abstract

**Purpose of Review:**

This paper provides an update from the literature on understanding of the relationship between cannabis and schizophrenia. In particular, the paper focuses on the latest findings and remaining areas that require investigation.

**Recent Findings:**

Three hypotheses have emerged as potential explanations for the association between cannabis and schizophrenia, namely cannabis can trigger schizophrenia, cannabis is used to mitigate symptoms of schizophrenia, and there are common factors which might account for the association. Biological and genetic factors dominate this field of research; this has been at the expense of exploring social and cultural contributory factors which influence cannabis and schizophrenia.

**Summary:**

The evidence for cannabis acting as a causal factor for schizophrenia has so far not been established. Research needs to extend beyond males drawn from western countries if we are to advance knowledge and understanding of the link between cannabis use and schizophrenia.

## Introduction

An estimated 192 million people used cannabis in 2016 demonstrating its status as the most popular drug after alcohol and tobacco globally [1]. As with all drugs, there are risks associated with its use; some risk is due to the substance and some will depend on the regulatory status of the drug in the local jurisdiction that the individual resides in. One risk of using cannabis continues to attract significant attention, namely the risk of developing psychosis. Distinguishing between causation and correlation on this issue has proved to be a challenge. However, it is critical that we understand how risks such as developing psychosis and schizophrenia for those that are exposed to cannabis unfold, so this knowledge could be used to reduce harm and to more effectively target scarce public health resources to high risk groups.

Scholars and clinicians have been aware of a link between cannabis and schizophrenia for over a century [2]. However, it wasn’t until cannabis use became popular in western countries during the 1960’s that researchers began to investigate the nature of the relationship. The seminal observational study by Andreasson and colleagues set the research agenda on this topic, reporting that cannabis was an independent risk factor for schizophrenia with 6% of heavy cannabis users going on to develop schizophrenia [3].

Three hypotheses outline the potential relationship between cannabis and schizophrenia in contemporary literature:

1. Cannabis can trigger schizophrenia in an individual who would not have developed the illness if they had not been exposed to the drug.
2. Individuals with a predisposition to schizophrenia use cannabis to mitigate the prodromal symptoms of schizophrenia, referred to as reverse causation.
3. Common cause suggests that other factors are responsible for the relationship such as childhood trauma or genetics for example.

Although the first of these hypotheses appears to be simple, it has proved to be difficult to investigate with confidence [4]. Research relies on observational methods mainly with all the limitations these have, but there are other problems in exploring this theory. It is widely thought that the dose of cannabis is a factor in increasing the risk of psychosis [5], but there is significant heterogeneity in the assessment of dose. Some researchers use exposure of 50 times or more, others use the proxy of last month usage to indicate regular use as opposed to infrequent or occasional use of the drug [6•]. This is compounded by the diagnostic criteria for cannabis-induced psychosis that rely on subjective assessment [7]. For instance, an assessing clinician or researcher trying to establish that cannabis played a causal role for an individual presenting with psychosis would need to decide how recent use of cannabis should be in order to make this differential diagnosis. The possibility for error is amplified when considering that metabolites of cannabis can be detected for weeks following exposure [8], clearly raising the risk of a false positive association and diagnosis of cannabis-induced psychosis.

The second hypothesis suggests individuals who are predisposed to schizophrenia are drawn to cannabis to mitigate the early signs of negative symptoms of schizophrenia. Although plausible, it should be stressed that several studies show how this is not the main reason these individuals consistently give as their motivation to use cannabis. Pleasure and recreation are the main reasons given by patients [9–11]. Although these reasons are clearly subjective and based on individual perception, they are important to understand particularly in the clinical setting as they provide an insight to the patients’ motivation for using cannabis even if this use is detrimental to their mental health.

An interesting and promising avenue of research is the potential that cannabis or rather the role a group of compounds found in cannabis might offer in treating schizophrenia. Cannabidiols have been shown to provide therapeutic value in the treatment of schizophrenia with a relatively low risk of adverse effects [12]. This might add credibility to the second hypothesis as individuals could be benefiting from using cannabis that contains these compounds. Mitigating factors here, however, include the emerging evidence that the composition of cannabis in recent years has been changing [12, 13•, 14]. These changes revolve around the ratios of Δ^9^-tetrahydrocannabinol (THC) and cannabidiols (CBD), some of the more prominent compounds in cannabis. Higher potency strains are higher in THC and lower in CBD.

This aspect is rarely assessed; even when potency or strength is investigated, the categories used to describe this factor are inconsistent and sometimes too generic to allow replication or translation. The increasing potency of cannabis that is available is thought to play a role in the risk of developing psychosis and other cannabis use disorders [15, 16]. This leads onto a further problem namely the qualifying period of experiencing psychosis that is required to qualify for a diagnosis of cannabis-induced psychosis. Some people will have a short-lived acute reaction to cannabis while others will go on to develop a longer-term problem. This offers the potential of over counting or under counting cases depending on where the individual presents to, how long they are assessed for and the depth and quality of the assessment conducted at that point in time.

The third hypothesis which proposes a common cause such as a shared genetic liability is responsible for the link between cannabis and schizophrenia [17, 18]. This has been explored more recently using sophisticated methods of analysis to try and untangle the relationship between cannabis and schizophrenia. One example used Mendelian randomisation to investigate the direction of the relationship in a study using a genome database [18]. This reported that there is a slightly increased risk of developing schizophrenia following initiation of cannabis with stronger evidence that schizophrenia predicts cannabis initiation.

So far, genetic orientated research has failed to provide a plausible explanation involving a purely biological or genetic factor that makes clear the direction of the relationship between cannabis and schizophrenia. Although it is possible that there is a relationship and even if that were to emerge as causal, it would be influenced significantly by the individual’s environment [19]. Most research in this field relies on animal studies which have their own limitations, but they still have a contribution to theoretical modelling.

## Knowledge Gaps

Beyond the aforementioned limitations, there are some other gaps in research and understanding of the relationship between cannabis and schizophrenia. The Sex and Gender Equity in Research (SAGER) guidelines acknowledge the inattention given to sex generally [20]. This appears to have limited our understanding of the links between cannabis and schizophrenia for women. The study by Andréasson included an all-male sample; research since then has either over-sampled males or failed to report differences by sex [21•]. Reporting sex in studies exploring cannabis and schizophrenia would not only improve our knowledge of the impact this has on females but may also provide useful information and insights for men [22]. If we had more information about the differences between the sexes in the risk of developing cannabis psychosis, this could prove useful in adding to knowledge about risk factors and who would benefit from targeted public health interventions to minimise the risk [23••].

Research into cannabis psychosis originates and continues to be dominated by participants drawn from western countries. Cannabis is used in most countries across the world although again we know more about use of the drug among western populations than any other. A recent review of the Indian literature investigating psychiatric problems including psychosis associated with cannabis use found a limited focus on aetiology and that all the studies bar one had all male samples [24]. This clearly leaves us with a skewed view of the impact on health resulting from use of cannabis. Drug use and its consequences are known to be sensitive to cultural influences; studies on cannabis psychosis have been dominated by samples drawn from America, Australia, and Europe. In some parts of Asia, there appears to be lower population use of cannabis which might in part account for the lower incidence of psychosis found in these populations [25].

Cannabis and tobacco are frequently used in combination so attention has turned to the synergistic effects of cannabis and tobacco on psychosis [26, 27]. Tobacco appears to be an independent risk factor for psychosis. As rates of cigarette smoking are elevated in those with psychosis compared with the general population there are some obvious general health benefits if cigarette use were to be reduced in this population [28]. As there are few modifiable risk factors for schizophrenia, there is a good case for trying to decouple tobacco from cannabis in those cannabis using populations as this could reduce the risk of psychosis by itself.

It is sometimes difficult for individuals to distinguish between cannabis and tobacco dependence particularly when they are used in combination. So even when someone has psychosis thought to have been associated with cannabis use, they can struggle to abstain from cannabis [29•]. Little evidence exists as to how this group can be encouraged and supported to abstain from cannabis use with the aim of reducing the impact of psychosis on their lives. There is the opportunity to learn from those with psychosis who have successfully reduced or abstained completely from using cannabis [30, 31].

## Conclusion

We need to carefully balance enquiry into cannabis and schizophrenia between neurophysiology and the social aspects of cannabis psychosis. Attention and research funding leans towards the biological hypothesis at the expense of the cultural [32••]. As cannabis use and the development of psychosis are both influenced by social as well as biological factors, it is important that we keep pursuing a balanced blend of enquiry.

Dose and frequency of cannabis use continue to be reliable indicators in the risk of developing psychosis [33]. However, some individuals appear to be sensitive to low levels of exposure to cannabis; it is still not clear whether that is due to increasing potency of cannabis or some other factor such as genetics.

Overall, we still have insufficient information and knowledge about who is at risk of developing cannabis psychosis prior to cannabis exposure to reliably produce a public health prevention strategy. As an increasing number of American states allow access to cannabis for recreational or medicinal purposes, this presents a large naturalistic experiment involving these populations, it will be some years before we can judge the impact of these regulatory changes on health and incidence and prevalence of psychosis in particular [34].
